# RNA Editing of Serotonin 2C Receptor and Alcohol Intake

**DOI:** 10.3389/fnins.2019.01390

**Published:** 2020-01-14

**Authors:** Masaki Tanaka, Yoshihisa Watanabe

**Affiliations:** ^1^Department of Anatomy and Neurobiology, Graduate School of Medical Science, Kyoto Prefectural University of Medicine, Kyoto, Japan; ^2^Department of Basic Geriatrics, Graduate School of Medical Science, Kyoto Prefectural University of Medicine, Kyoto, Japan

**Keywords:** 5-HT_2__C_R, RNA editing, alcohol intake, nucleus accumbens, mice

## Abstract

Serotonin 2C receptor (5-HT_2__C_R) belongs to the superfamily of seven transmembrane domain receptors coupled to G proteins (GPCR). It is broadly distributed in the CNS and its expression is relatively high in the limbic system including the amygdala, nucleus accumbens (NAc), hippocampus, and hypothalamus. Based on its expression patterns and numerous pharmacological studies, 5-HT_2__C_R is thought to be involved in various brain functions including emotion, appetite, and motor behavior. Here, we review 5-HT_2__C_R and its relationship with alcohol intake with a particular focus on the involvement of 5-HT_2__C_R mRNA editing and its association with alcohol preference in mice. RNA editing is a post-transcriptional modification mechanism. In mammals, adenosine is converted to inosine by the deamination enzymes ADAR1 and ADAR2. 5-HT_2__C_R is the only GPCR subjected to RNA editing within the coding region. It has five editing sites in exon 5 that encode the second intracellular loop. Consequently, three amino acids residues (I156, N158, and I160) of the unedited receptor (INI) may be altered to differently edited isoforms, resulting in a change of receptor activity such as 5-HT potency and G-protein coupling. 5-HT_2__C_R in the NAc is involved in enhanced alcohol drinking after chronic alcohol exposure and alterations in 5-HT_2__C_R mRNA editing is important in determining the alcohol preference using different strains of mice and genetically modified mice. RNA editing of this receptor may participate in the development of alcoholism.

## Introduction

The serotonin 2C receptor (5-HT_2__C_R) is a member of the 5-HT receptor family, which is divided into seven groups from 5-HT_1_R to 5-HT_7_R (Hoyer et al., 1994). 5-HT is produced in neurons located in specific areas of the brainstem that project axons throughout the central nervous system (CNS) (Dahlstroem and Fuxe, 1964). 5-HT acts as a neurotransmitter via receptors and it is involved in the regulation of emotional control, sleep, appetite, and learning. Many studies have reported the roles of 5-HT in psychiatric disorders such as depression and schizophrenia (Mohammad-Zadeh et al., 2008). The seven 5-HT receptors are further divided into at least 14 subgroups (Barnes and Sharp, 1999). In this review article we describe the general aspects of 5-HT_2__C_R, its mRNA editing mechanism, and the relationship between 5-HT_2__C_R and alcohol intake, particularly alterations in 5-HT_2__C_R mRNA editing and alcohol drinking behavior.

## 5-HT_2__C_R

5-HT_2__C_R belongs to the family of seven transmembrane G protein coupled receptors (GPCRs). Although it has a similar binding activity to 5-HT in 5-HT_1__A_R and 5-HT_1__B_R, it forms a family with 5-HT_2__A_R and 5-HT_2__B_R due to the similarity of their amino acid sequences. 5-HT_2__C_R has 57% amino acid identity with 5-HT_2__A_R (Hoyer et al., 2002). The *5-HT_2__C_R* gene (*HTR2C*) is located on chromosome Xq24 and harbors multiple introns within its coding regions (Milatovich et al., 1992). In addition to splicing variants, *5-HT_2__C_R* pre-mRNA undergoes RNA editing at five sites (Fitzgerald et al., 1999; Werry et al., 2008; Chagraoui et al., 2016).

The distribution of 5-HT_2__C_R in the brain indicates its role in a variety of functions. Radioautography by tritium labeling, immunohistochemistry and *in situ* hybridization have revealed that 5-HT_2__C_R is widely expressed in the CNS but not in the peripheral nervous system (Werry et al., 2008). In the CNS, it is more broadly expressed than 5-HT_2__A_R and 5-HT_2__B_R. It is strongly expressed in the choroid plexus along the cerebral ventricle (Sanders-Bush and Breeding, 1988), followed by the prefrontal cortex, basal ganglia (caudate nucleus and substantia nigra), and limbic system including the anterior olfactory nucleus, lateral habenular nucleus, hippocampus, amygdala, cingulate cortex, nucleus accumbens, ventral tegmental area (VTA) and hypothalamus in rats (Clemett et al., 2000; Li et al., 2004). In humans, 5-HT_2__C_R was reported to be expressed in the cerebral cortex, cerebellum, and substantia nigra (Pasqualetti et al., 1999). Regarding its intracellular localization, 5-HT_2__C_R is mainly present in the post-synaptic membrane, but in some brain regions it is expressed in the presynaptic membrane (Becamel et al., 2004). Its distribution in specific brain regions and pharmacological studies using agonists and antagonists of 5-HT_2__C_R revealed it has a role in emotion and hypothalamic function. If the physiological functions of this receptor are disturbed, various diseases such as anxiety, depression, addiction, obesity, and epilepsy may develop (Di Giovanni and De Deurwaerdere, 2016; Palacios et al., 2017). Analyses using *HTR2C* gene knockout mice also supports this idea (Tecott et al., 1995; Chou-Green et al., 2003). 5-HT_2__C_R couples with Gq/11, Gα12/13, and Gαi and regulates pathways at the second messenger level via inositol-3-phosphate, Ca^2+^, cAMP, and arachidonic acid. In addition, the activation of cGMP, ERK1/2 and protein kinase C were also reported to act as second messengers (Berg et al., 1994; Werry et al., 2008). These findings support the idea that a disturbance in one or more of these pathways may cause the development of diseases related to 5-HT_2__C_R. Therefore, drug design studies have targeted this receptor. However, this is not simple because 5-HT_2__C_R has constitutive activity in the absence of ligand binding and amino acid changes occur due to mRNA editing and alternative splicing. It was recently reported that the truncated isoform of 5-HT_2__C_R generated by alternative slicing heterodimerizes with full length 5-HT_2__C_R intracellularly to decrease receptor signaling (Martin et al., 2013; Zhang et al., 2016; Stamm et al., 2017). It was reported that 5-HT2CR also dimerizes with other receptors, which impacts subsequent signaling (Schellekens et al., 2013, 2015).

## RNA Editing of 5-HT_2__C_R mRNA

A variety of gene products occurs by post-transcriptional modification even in the same genome. Most well-known alternative splicing occurs in more than 70% of mammalian genes (Maas et al., 2006). Another modification is mRNA editing. In vertebrates, adenosine of RNA is deaminated to inosine by adenosine deaminase enzymes acting on RNA (ADAR). Inosine is regarded as guanosine when RNA is transcribed because of its structural similarity. ADARs specifically catalyze double strand RNAs. To date, ADAR1, ADAR2, and ADAR3 have been identified, whereas target RNAs of ADAR3 have not been found (Nishikura, 2010). RNA is an energetically unstable molecule, thus it is considered that RNA is edited in order to respond rapidly to a change in the surrounding environment (Tohda, 2014). Most RNA editing occurs in 3′ or 5′ untranslated regions and this regulates gene expression. Less than 30 genes undergo mRNA editing within coding regions (Nishikura, 2010). Human ENCODE RNA-seq data indicate that only 123 editing sites are present in protein-coding sequences (Park et al., 2012). In these cases, a different isoform can be produced after RNA editing. The majority of genes that undergo mRNA editing within exons are ion channels or receptors of neurotransmitters. Among them, two neurotransmitter receptors in the CNS, GluR2/GluA2, a subunit of the AMPA type glutamatergic receptor and 5-HT_2__C_R, a GPCR, have been intensively analyzed. GluR2/GluA2, which regulates Ca^2+^ influx into the cell, undergoes editing at two sites. Glutamine (Q) is substituted to arginine (R) by ADAR2 at the Q/R site and arginine is substituted to glycine (G) by ADAR1 and ADAR2 at the R/G site. Usually the Q/R site of GluR2 is edited 100% to inhibit the Ca^2+^ influx; however, when the editing frequency is decreased, the permeability of Ca^2+^ into the cell is increased causing neuronal cell death. A decrease of RNA editing at the Q/R site of GluR2 in motor neurons in the anterior horn of the spinal cord was suggested to cause amyotrophic lateral sclerosis (Kwak et al., 2010).

**FIGURE 1:**
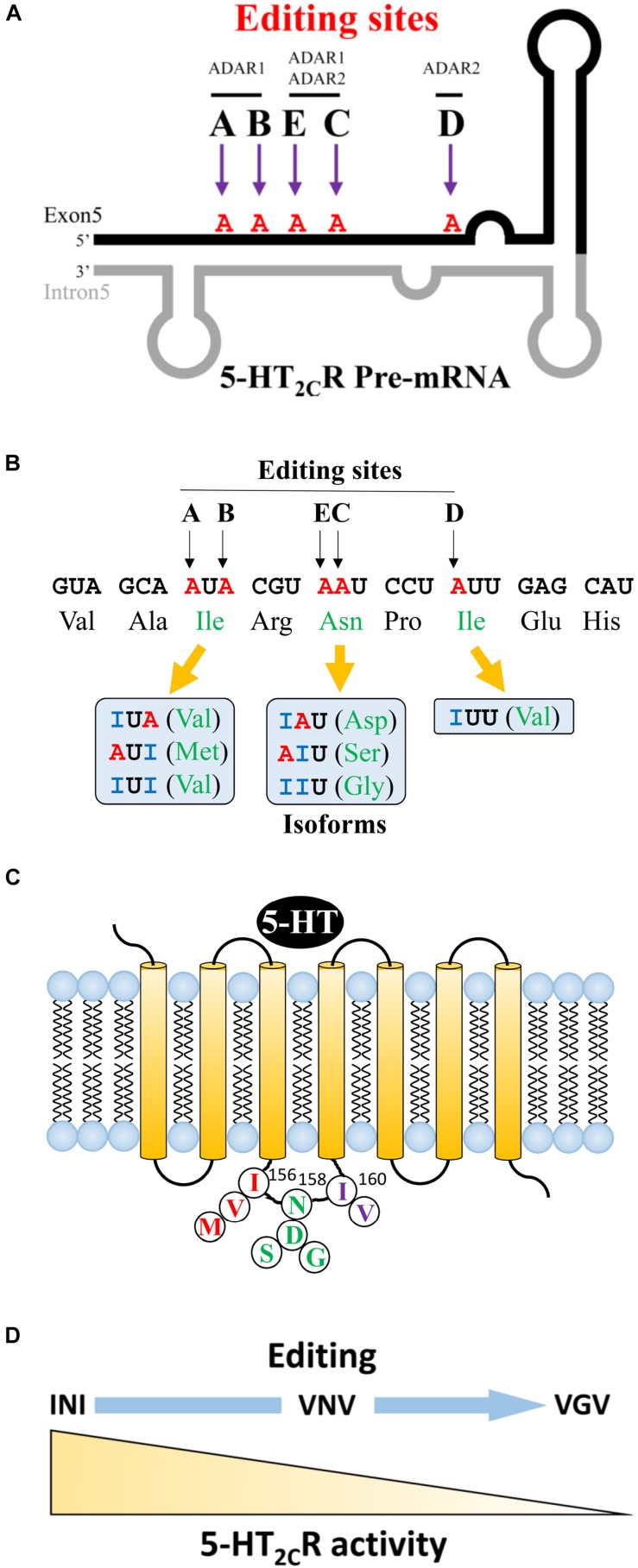
Editing sites at 5-HT_2__C_R pre-mRNA and various amino acid sequences. **(A)** Five sites (A to E) in exon 5 of 5-HT_2__C_R mRNA. Adenosine to Inosine (A to I) RNA editing occurs in the region where exon 5 makes a double strand with intron 5. Editing enzyme ADAR1 acts on A and B sites, whereas ADAR2 acts on D sites. E and C sites are edited by both ADAR1 and ADAR2. **(B)** The structure of 5-HT_2__C_R has seven transmembrane regions. Three amino acids (156, 158, 160) in the second intracellular loop may be edited by RNA editing. **(C)** The variation of edited isoforms. From A and B editing sites, non-edited isoleucine (Ile) can be edited to valine (Val) or methionine (Met). Asparagine (An) in E and C sites may be edited to aspartic acid (Asp), serine (Ser), or glycine (Gly). Amino acid isoleucine at the D site may be edited to valine (Val). **(D)** 5-HT_2__C_R activity is reduced in relation to an increase in RNA editing.

Regarding 5-HT_2__C_R, adenosine to inosine editing can occur at five sites (A–E) in exon 5, which encodes a second intracellular loop. The A and B sites are catalyzed by ADAR1 and the D site is catalyzed by ADAR2 (Figure 1A). The C and E sites are edited by ADAR1 and 2. The second intracellular loop is an important region for coupling to G proteins, which affects downstream signaling cascades. The presence or absence of editing at each of the five sites results in changes in three amino acid sequences at 156 (isoleucine, I), 158 (asparagine, N), and 160 (isoleucine, I) (Figure 1B). When mRNA editing occurs at A and B sites of the 156 non-edited isoform, isoleucine may change to valine (V) or methionine (M). At C and E sites, 158 asparagine may change to aspartic acid (D), serine (S), or glycine (G). At the D site, 160 isoleucine can be substituted to valine (V). If editing happens at all sites, a VGV type isoform is generated. Thus, from the non-edited INI isoform, 24 isoforms can be produced theoretically (Figure 1C) (Wang et al., 2000; Werry et al., 2008). 5-HT_2__C_R has its own constitutive activity in the absence of ligand binding. The unedited isoform INI has the highest constitutive activity, which is downregulated in edited isoforms (Herrick-Davis et al., 1999; Niswender et al., 1999). Moreover, the sensitivity and binding affinity to 5-HT varies dependent on the specific isoform (Burns et al., 1997; Fitzgerald et al., 1999; Berg et al., 2001; Gurevich et al., 2002). We previously examined the activity of each isoform *in vitro* by measuring the constitutive activity and inositol phosphate productivity after 5-HT stimulation. We observed that the non-edited INI isoform had the highest activities and that the all-edited VGV isoform had the lowest activities (Figure 1D) (Watanabe et al., 2014). Regarding the amount of each isoform, differences among species and brain regions were reported. The most abundant isoform in the whole brain was the VSV isoform in humans and the VNV isoform in rats. In humans, VSV is the major isoform in the thalamus, hypothalamus, and amygdala; however, ISV and VSI are the major isoforms in the cerebellum and hippocampus, respectively (Wang et al., 2000). Recently, it was reported that VSV was the predominant isoform in many regions except INI in the cerebellum of the porcine brain (Larsen et al., 2016). We examined the isoforms in three mouse strains: VNV was the most abundant in the nucleus accumbens (NAc), dorsal raphe nucleus (DRN) and amygdala (Hackler et al., 2006). The second most common isoform was VSV in the NAc and VNI in the DRN suggesting regional differences (Wang et al., 2000).

Taken together, these findings suggest that 5-HT_2__C_R editing may be involved in emotional and psychiatric disorders. Patients with these disorders might have an altered frequency of 5-HT_2__C_R RNA editing. Indeed, many studies have reported that 5-HT_2__C_R RNA editing was altered in patients with major depression or in suicide victims (Niswender et al., 2001; Gurevich et al., 2002; Iwamoto and Kato, 2003; Dracheva et al., 2008; Simmons et al., 2010; Di Narzo et al., 2014; Weissmann et al., 2016). In schizophrenia patients, 5-HT_2__C_R RNA editing was reduced in the frontal cortex (Sodhi et al., 2001). Anxiety and stress conditions also may be related to 5-HT_2__C_R RNA editing (Englander et al., 2005; Mombereau et al., 2010; Bombail et al., 2014). In addition to mental distress, editing changes in 5-HT_2__C_R mRNA were reported to be involved in obesity, and spinal cord and peripheral nerve injury (Morabito et al., 2010; Schellekens et al., 2012; Uchida et al., 2017).

Recently, the gut microbiota was reported to influence 5-HT_2__C_R mRNA editing levels during development in mouse brain (van de Wouw et al., 2019). Early life stress related to maternal separation induced increased depression-like behavior and 5-HT_2__C_R RNA editing during mouse adulthood (Bhansali et al., 2007). Moreover, alterations in editing might occur trans-generationally. It was reported that chronic unpredictable stress in pre-reproductive female rats affected the 5-HT_2__C_R RNA editing in two generations of offspring (Zaidan et al., 2018). Maternal treatment with a serotonin-specific reuptake inhibitor (SSRI), fluoxetine, after stress reversed the effect of these editing changes in the prefrontal cortex and amygdala of new born offspring (Zaidan and Gaisler-Salomon, 2015). Collectively, environmental conditions that affect the editing of 5-HT_2__C_R mRNA with its receptor function might be therapeutic targets of disease.

## Alcohol Drinking Behavior and 5-Ht_2__C_R

It is generally thought that alcohol is consumed for its positive reinforcing effects and that chronic exposure to alcohol results in adaptations with abnormal drinking patterns. The mesolimbic dopaminergic projections from the VTA to the NAc in the midbrain have been implicated in playing an essential role in the brain reward system (Engel and Jerlhag, 2014). Dopaminergic dysfunction in the NAc caused by chronic alcohol consumption is involved in alcoholism (Heinz, 2002). One of the modulating factors of this VTA-NAc dopaminergic system is 5-HT from neurons of the DRN (Yoshimoto and McBride, 1992). 5-HT stimulates the alcohol-induced excitation of VTA neurons (Brodie et al., 1995). Chronic alcohol exposure affects serotonergic synaptic transmission and causes adaptive changes in its receptors. 5-HT_2__C_R appears to undergo such adaptive changes (Pandey et al., 1995; Lovinger, 1997). Treatment of the NAc with a 5-HT_2__C_R antagonist inhibited alcohol-induced behavioral sensitization in mice (Andrade et al., 2011). We previously reported that among 5-HT receptors, 5-HT_2__C_R in the NAc was involved in increased alcohol drinking behavior of C57BL/6J mice after chronic alcohol exposure (Yoshimoto et al., 2012). We developed a chronic alcohol exposure animal model via the inhalation of vapored ethanol. After chronic exposure to alcohol, mice had a higher alcohol intake compared with control animals, whereas their water consumption was similar to that of the control group. These mice had an enhanced expression of 5-HT_2__C_R at the mRNA and protein levels in the NAc. The expression of 5-HT_7_R mRNA in the NAc was also increased; however, only systemic treatment with a specific 5-HT_2__C_R antagonist or intra NAc treatment inhibited the enhanced alcohol intake after chronic alcohol exposure (Yoshimoto et al., 2012).

**FIGURE 2:**
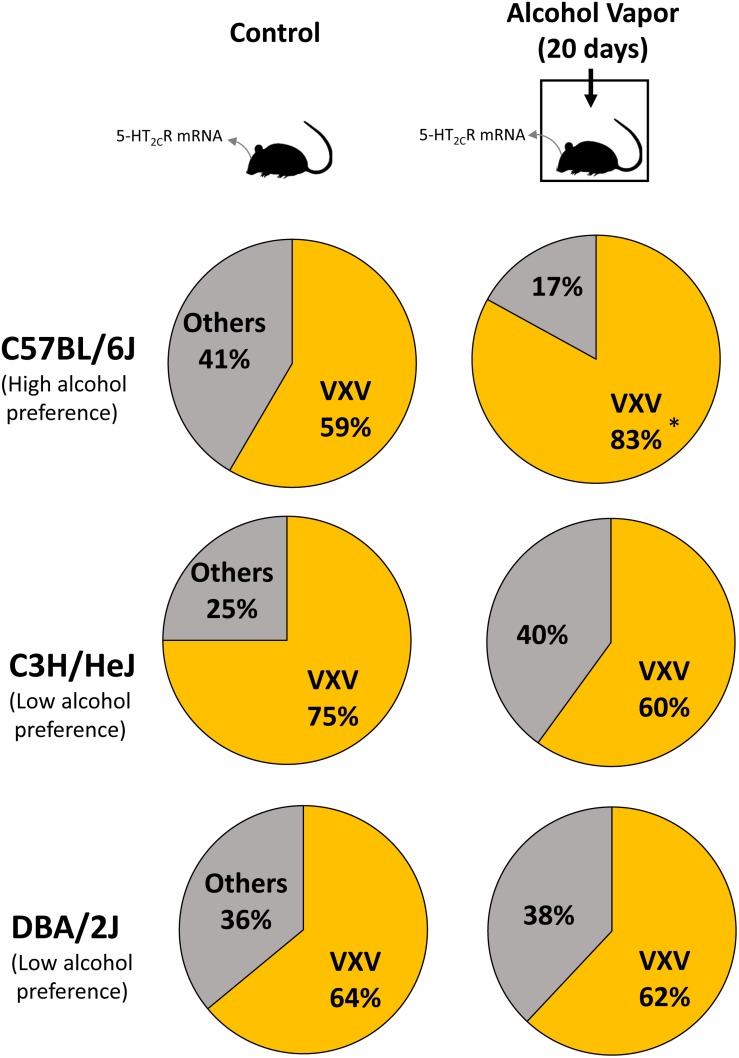
Mice were intermittently exposed to alcohol by inhalation for a period of 20 days. Frequencies of 5-HT_2__C_R mRNA editing in control **(left)** and chronic alcohol exposed mice **(right)** were measured by the cloning–sequencing analysis of RT-PCR products from the NAc. Pie charts of the ratio of edited isoforms, V-X-V (at least two amino acids are edited to valine) in three strains of mice after chronic alcohol exposure. C57BL/6J mice, but not C3H/HeJ and DBA/2J mice, had increased VXV type isoforms after alcohol intake. Statistical analyses were performed using Fisher’s exact test. Two-sided tests were used to calculate *P*-values. ^∗^*P* < 0.01.

Previous studies reported differences in alcohol preference among mouse inbred strains. C57BL/6J, but not C3H/HeJ and DBA/2J mice, drank more alcohol after alcohol exposure compared with controls (Yoshimoto and Komura, 1989). The expression of 5-HT_2__C_R mRNA was increased in the NAc of C57BL/6J mice but in C3H/HeJ or DBA/2J mice. As 5-HT_2__C_R is subjected to pre mRNA editing, we examined the editing frequency of 5-HT_2__C_R mRNA. In C57BL/6J mice, edited isoforms of 5-HT_2__C_R were increased in the NAc but not the hippocampus. Particularly, VXV isoforms such as VGV, VNV, VSV, and VDV in which the first (156) and third (160) of three replaceable amino acids were edited to valine, were increased in C57BL/6J mice after chronic alcohol exposure; however, these increases were not observed in C3H/HeJ or DBA/2J mice (Figure 2 and Table 1). The editing enzymes ADAR1 and ADAR2 were increased in the NAc of C57BL/6J mice after chronic alcohol exposure but not in C3H/HeJ or DBA/2J mice. Taken together, C57BL/6J mice showed enhanced alcohol intake after chronic alcohol exposure related to the increased RNA editing of 5-HT_2__C_R; however, this was not observed in C3H/HeJ or DBA/2J mice that did not show enhanced alcohol intake. From this result, alterations in the RNA editing of 5-HT_2__C_R may underlie alcohol preference. Next, we examined mice that exclusively expressed the non-edited INI isoform of 5-HT_2__C_R (Kawahara et al., 2008) and compared them with wild type littermates on the C57BL/6J background. ADARs recognize double-stranded RNA. Exon 5 of the 5-HT_2__C_R mRNA consists of an imperfect double-stranded RNA with intron 5. Intron 5 was deleted in INI knock-in mice to prevent editing by ADARs at five sites in exon 5. INI mice had a similar phenotype of food intake, water intake and weight gain as wild type mice. We examined alcohol consumption in INI and wild type mice after chronic alcohol exposure and observed that wild type mice had an increase in alcohol intake; however, INI mice had a similar level of alcohol intake to the controls even on the C57BL/6J background. This result indicates that the editing of 5-HT_2__C_R mRNA underlies the increase in alcohol consumption after chronic alcohol exposure in mice. The importance of RNA editing in alcohol preference was confirmed using non-changing RNA editing (INI) mice. The constitutive activity of 5-HT_2__C_R inhibits accumbal dopamine release (De Deurwaerdere et al., 2004; Di Matteo et al., 2004). 5-HT_2__C_R in the NAc is expressed in GABAergic neurons as well as in the VTA, DRN, and medial prefrontal cortex (Bubar et al., 2011; Spoida et al., 2014; Nocjar et al., 2015; Aoki et al., 2016). It was reported that the GABA neuronal system was also involved in alcohol reward and dependence (Koob et al., 1998). Increased edited isoforms of 5-HT_2__C_R with low signaling induced by chronic alcohol exposure may enhance dopamine release by modulating GABAergic neurons in the NAc. Consequently, mice may develop increased alcohol consumption. Although the mesolimbic dopamine system is modulated by 5-HT_2__C_R (Di Giovanni and De Deurwaerdere, 2016; De Deurwaerdere and Di Giovanni, 2017) and its RNA editing seems to affect the addiction to drugs, few studies have investigated the relationship between them. Cocaine administration to the rat cerebral cortex for 7 days did not alter the RNA editing of 5-HT_2__C_R in the cerebral cortex, hippocampus or midbrain (Iwamoto and Kato, 2002). Nicotine withdrawal reduced editing at the E site in the hippocampus of rats (Zaniewska et al., 2015). Further studies are necessary to reveal the role of RNA editing of 5-HT_2__C_R in drug addiction in the future.

**TABLE 1:** Frequencies of 5-HT_2__C_R isoforms in C57BL/6J mice.

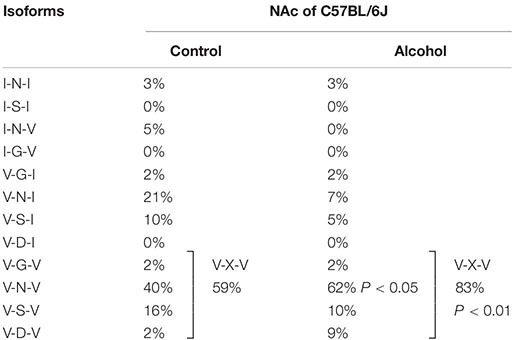

*Statistical analyses were performed by Fisher’s exact test. Two-sided tests were used to calculate *P*-values. Reproduced partly with permission by Oxford University Press (Watanabe et al., 2014).*

Among each of the editing sites (A–E) of 5-HT_2__C_R in the NAc, the editing frequency at the D site, an ADAR2 specific site, was significantly increased in C57BL/6J mice after chronic alcohol exposure. ADAR2 expression was enhanced as well as ADAR1 in the NAc (Watanabe et al., 2014). Regarding RNA editing in the NAc, GluA2 RNA editing at the Q/R site in the NAc was reduced by forced cocaine abstinence, and ADAR2 overexpression in the NAc attenuated cocaine-seeking behavior (Schmidt et al., 2015). We examined the involvement of RNA editing in alcohol drinking by deleting ADAR2 in the NAc using conditional ADAR2 knockout mice (ADAR2*^*flox*^*^/^*^*flox*^*) on a C57BL/6J genetic background (Hideyama et al., 2010). Adeno-associated virus (AAV)-green fluorescent protein (GFP)/Cre into the NAc of ADAR2*^*flox*^*^/^*^*flox*^* mice was used to specifically delete the ADAR2 gene. Accumbal RNA editing frequency in the ADAR2-dependent editing sites of GluA2 Q/R, 5-HT_2__C_R site D and CYFIP2 K/E, was significantly reduced (Shirahase et al., 2018). In contrast to wild type mice, ADAR2 KO mice did not develop enhanced ethanol intake or ethanol preference after chronic exposure to ethanol vapor (Shirahase et al., 2018). ADAR2 mediates the RNA editing of various ion channels and receptors such as the Ca_V_1.3 calcium ion channel, K_V_1.1 potassium ion channel, 5-HT_2__C_R, GluA2, and GABA_A_ (Bhalla et al., 2004; Bazzazi et al., 2013; Behm and Ohman, 2016). Therefore, other receptors as well as 5-HT_2__C_R may be involved in the alcohol drinking behavior of this model by a NAc-specific reduction of ADAR2 expression. Increased cortical expression of ADAR2 and 5-HT_2__C_R mRNA editing was reported in major depressive suicide victims (Simmons et al., 2010). ADAR2 is highly expressed in the brain and its degradation is regulated by E3 ubiquitin ligase WWP2 (Marcucci et al., 2011; Gallo et al., 2017). Therefore, control of the ADAR2 level in the NAc might be a target for the development of treatment for alcoholism.

## Conclusion

We reviewed the general features of 5-HT_2__C_R and its mRNA editing with specific reference to alcohol preference. The accumbal expression and mRNA editing of 5-HT_2__C_R is involved in alcohol intake in mice and this mechanism may be also relevant to human alcoholism. The regulation of 5-HT_2__C_R RNA editing might be a new therapeutic strategy for alcohol addiction.

## Author Contributions

MT conceived the review and wrote the manuscript. YW prepared figures and table.

## Conflict of Interest

The authors declare that the research was conducted in the absence of any commercial or financial relationships that could be construed as a potential conflict of interest.

## References

[B1] Andrade A. L. Abrahao K. P. Goeldner F. O. Souza-Formigoni M. L. (2011). Administration of the 5-HT2C receptor antagonist SB-242084 into the nucleus accumbens blocks the expression of ethanol-induced behavioral sensitization in Albino Swiss mice. *Neuroscience* 189 178–186. 10.1016/j.neuroscience.2011.05.028 21658435

[B2] Aoki M. Watanabe Y. Yoshimoto K. Tsujimura A. Yamamoto T. Kanamura N. (2016). Involvement of serotonin 2C receptor RNA editing in accumbal neuropeptide Y expression and behavioural despair. *Eur. J. Neurosci.* 43 1219–1228. 10.1111/ejn.13233 26950265

[B3] Barnes N. M. Sharp T. (1999). A review of central 5-HT receptors and their function. *Neuropharmacology* 38 1083–1152. 10.1016/s0028-3908(99)00010-6 10462127

[B4] Bazzazi H. Ben Johny M. Adams P. J. Soong T. W. Yue D. T. (2013). Continuously tunable Ca(2+) regulation of RNA-edited Ca_V_1.3 channels. *Cell Rep.* 5 367–377. 10.1016/j.celrep.2013.09.006 24120865 PMC4349392

[B5] Becamel C. Gavarini S. Chanrion B. Alonso G. Galeotti N. Dumuis A. (2004). The serotonin 5-HT2A and 5-HT2C receptors interact with specific sets of PDZ proteins. *J. Biol. Chem*. 279 20257–20266. 14988405 10.1074/jbc.M312106200

[B6] Behm M. Ohman M. (2016). RNA editing: a contributor to neuronal dynamics in the mammalian brain. *Trends Genet.* 32 165–175. 10.1016/j.tig.2015.12.005 26803450

[B7] Berg K. A. Clarke W. P. Sailstad C. Saltzman A. Maayani S. (1994). Signal transduction differences between 5-hydroxytryptamine type 2A and type 2C receptor systems. *Mol. Pharmacol.* 46 477–484. 7935328

[B8] Berg K. A. Cropper J. D. Niswender C. M. Sanders-Bush E. Emeson R. B. Clarke W. P. (2001). RNA-editing of the 5-HT(2C) receptor alters agonist-receptor-effector coupling specificity. *Br. J. Pharmacol.* 134 386–392. 10.1038/sj.bjp.0704255 11564657 PMC1572953

[B9] Bhalla T. Rosenthal J. J. Holmgren M. Reenan R. (2004). Control of human potassium channel inactivation by editing of a small mRNA hairpin. *Nat. Struct. Mol. Biol.* 11 950–956. 10.1038/nsmb825 15361858

[B10] Bhansali P. Dunning J. Singer S. E. David L. Schmauss C. (2007). Early life stress alters adult serotonin 2C receptor pre-mRNA editing and expression of the alpha subunit of the heterotrimeric G-protein G q. *J. Neurosci.* 27 1467–1473. 10.1523/JNEUROSCI.4632-06.2007 17287521 PMC6673584

[B11] Bombail V. Qing W. Chapman K. E. Holmes M. C. (2014). Prevention of 5-hydroxytryptamine2C receptor RNA editing and alternate splicing in C57BL/6 mice activates the hypothalamic-pituitary-adrenal axis and alters mood. *Eur. J. Neurosci.* 40 3663–3673. 10.1111/ejn.12727 25257581 PMC4282755

[B12] Brodie M. S. Trifunovic R. D. Shefner S. A. (1995). Serotonin potentiates ethanol-induced excitation of ventral tegmental area neurons in brain slices from three different rat strains. *J. Pharmacol. Exp. Ther.* 273 1139–1146. 7791084

[B13] Bubar M. J. Stutz S. J. Cunningham K. A. (2011). 5-HT(2C) receptors localize to dopamine and GABA neurons in the rat mesoaccumbens pathway. *PLoS One* 6:e20508. 10.1371/journal.pone.0020508 21687728 PMC3110193

[B14] Burns C. M. Chu H. Rueter S. M. Hutchinson L. K. Canton H. Sanders-Bush E. (1997). Regulation of serotonin-2C receptor G-protein coupling by RNA editing. *Nature* 387 303–308. 10.1038/387303a0 9153397

[B15] Chagraoui A. Thibaut F. Skiba M. Thuillez C. Bourin M. (2016). 5-HT2C receptors in psychiatric disorders: a review. *Prog. Neuropsychopharmacol. Biol. Psychiatry* 66 120–135. 10.1016/j.pnpbp.2015.12.006 26739950

[B16] Chou-Green J. M. Holscher T. D. Dallman M. F. Akana S. F. (2003). Repeated stress in young and old 5-HT(2C) receptor knockout mice. *Physiol. Behav.* 79 217–226. 10.1016/s0031-9384(03)00096-9 12834793

[B17] Clemett D. A. Punhani T. Duxon M. S. Blackburn T. P. Fone K. C. (2000). Immunohistochemical localisation of the 5-HT2C receptor protein in the rat CNS. *Neuropharmacology* 39 123–132. 10.1016/s0028-3908(99)00086-6 10665825

[B18] Dahlstroem A. Fuxe K. (1964). Evidence for the existence of monoamine-containing neurons in the central nervous system. I. Demonstration of monoamines in the cell bodies of brain stem neurons. *Acta Physiol. Scand. Suppl.* 232 1–55. 14229500

[B19] De Deurwaerdere P. Di Giovanni G. (2017). Serotonergic modulation of the activity of mesencephalic dopaminergic systems: therapeutic implications. *Prog. Neurobiol.* 151 175–236. 10.1016/j.pneurobio.2016.03.004 27013075

[B20] De Deurwaerdere P. Navailles S. Berg K. A. Clarke W. P. Spampinato U. (2004). Constitutive activity of the serotonin2C receptor inhibits in vivo dopamine release in the rat striatum and nucleus accumbens. *J. Neurosci.* 24 3235–3241. 10.1523/jneurosci.0112-04.2004 15056702 PMC6730027

[B21] Di Giovanni G. De Deurwaerdere P. (2016). New therapeutic opportunities for 5-HT2C receptor ligands in neuropsychiatric disorders. *Pharmacol. Ther.* 157 125–162. 10.1016/j.pharmthera.2015.11.009 26617215

[B22] Di Matteo V. Pierucci M. Esposito E. (2004). Selective stimulation of serotonin2c receptors blocks the enhancement of striatal and accumbal dopamine release induced by nicotine administration. *J. Neurochem.* 89 418–429. 10.1111/j.1471-4159.2004.02337.x 15056285

[B23] Di Narzo A. F. Kozlenkov A. Roussos P. Hao K. Hurd Y. Lewis D. A. (2014). A unique gene expression signature associated with serotonin 2C receptor RNA editing in the prefrontal cortex and altered in suicide. *Hum. Mol. Genet.* 23 4801–4813. 10.1093/hmg/ddu195 24781207 PMC4140462

[B24] Dracheva S. Patel N. Woo D. A. Marcus S. M. Siever L. J. Haroutunian V. (2008). Increased serotonin 2C receptor mRNA editing: a possible risk factor for suicide. *Mol. Psychiatry* 13 1001–1010. 10.1038/sj.mp.4002081 17848916

[B25] Engel J. A. Jerlhag E. (2014). Alcohol: mechanisms along the mesolimbic dopamine system. *Prog. Brain Res.* 211 201–233. 10.1016/B978-0-444-63425-2.00009-X 24968782

[B26] Englander M. T. Dulawa S. C. Bhansali P. Schmauss C. (2005). How stress and fluoxetine modulate serotonin 2C receptor pre-mRNA editing. *J. Neurosci.* 25 648–651. 10.1523/jneurosci.3895-04.2005 15659601 PMC6725319

[B27] Fitzgerald L. W. Iyer G. Conklin D. S. Krause C. M. Marshall A. Patterson J. P. (1999). Messenger RNA editing of the human serotonin 5-HT2C receptor. *Neuropsychopharmacology* 21(Suppl. 2), 82S–90S. 10.1016/s0893-133x(99)00004-4 10432493

[B28] Gallo A. Vukic D. Michalik D. O’Connell M. A. Keegan L. P. (2017). ADAR RNA editing in human disease; more to it than meets the I. *Hum. Genet.* 136 1265–1278. 10.1007/s00439-017-1837-0 28913566

[B29] Gurevich I. Englander M. T. Adlersberg M. Siegal N. B. Schmauss C. (2002). Modulation of serotonin 2C receptor editing by sustained changes in serotonergic neurotransmission. *J. Neurosci.* 22 10529–10532. 10.1523/jneurosci.22-24-10529.2002 12486144 PMC6758441

[B30] Hackler E. A. Airey D. C. Shannon C. C. Sodhi M. S. Sanders-Bush E. (2006). 5-HT(2C) receptor RNA editing in the amygdala of C57BL/6J, DBA/2J, and BALB/cJ mice. *Neurosci. Res.* 55 96–104. 10.1016/j.neures.2006.02.005 16580757

[B31] Heinz A. (2002). Dopaminergic dysfunction in alcoholism and schizophrenia–psychopathological and behavioral correlates. *Eur. Psychiatry* 17 9–16. 10.1016/s0924-9338(02)00628-4 11918987

[B32] Herrick-Davis K. Grinde E. Niswender C. M. (1999). Serotonin 5-HT2C receptor RNA editing alters receptor basal activity: implications for serotonergic signal transduction. *J. Neurochem.* 73 1711–1717. 10.1046/j.1471-4159.1999.731711.x 10501219

[B33] Hideyama T. Yamashita T. Suzuki T. Tsuji S. Higuchi M. Seeburg P. H. (2010). Induced loss of ADAR2 engenders slow death of motor neurons from Q/R site-unedited GluR2. *J. Neurosci.* 30 11917–11925. 10.1523/JNEUROSCI.2021-10.2010 20826656 PMC6633551

[B34] Hoyer D. Clarke D. E. Fozard J. R. Hartig P. R. Martin G. R. Mylecharane E. J. (1994). International union of pharmacology classification of receptors for 5-hydroxytryptamine (Serotonin). *Pharmacol. Rev.* 46 157–203. 7938165

[B35] Hoyer D. Hannon J. P. Martin G. R. (2002). Molecular, pharmacological and functional diversity of 5-HT receptors. *Pharmacol. Biochem. Behav.* 71 533–554. 10.1016/s0091-3057(01)00746-8 11888546

[B36] Iwamoto K. Kato T. (2002). Effects of cocaine and reserpine administration on RNA editing of rat 5-HT2C receptor estimated by primer extension combined with denaturing high-performance liquid chromatography. *Pharmacogenomics J.* 2 335–340. 10.1038/sj.tpj.6500130 12439740

[B37] Iwamoto K. Kato T. (2003). RNA editing of serotonin 2C receptor in human postmortem brains of major mental disorders. *Neurosci. Lett.* 346 169–172. 10.1016/s0304-3940(03)00608-6 12853111

[B38] Kawahara Y. Grimberg A. Teegarden S. Mombereau C. Liu S. Bale T. L. (2008). Dysregulated editing of serotonin 2C receptor mRNAs results in energy dissipation and loss of fat mass. *J. Neurosci.* 28 12834–12844. 10.1523/jneurosci.3896-08.2008 19036977 PMC2615198

[B39] Koob G. F. Roberts A. J. Schulteis G. Parsons L. H. Heyser C. J. Hyytia P. (1998). Neurocircuitry targets in ethanol reward and dependence. *Alcohol. Clin. Exp. Res.* 22 3–9. 10.1111/j.1530-0277.1998.tb03611.x 9514280

[B40] Kwak S. Hideyama T. Yamashita T. Aizawa H. (2010). AMPA receptor-mediated neuronal death in sporadic ALS. *Neuropathology* 30 182–188. 10.1111/j.1440-1789.2009.01090.x 20102521

[B41] Larsen K. Momeni J. Farajzadeh L. Bendixen C. (2016). Differential A-to-I RNA editing of the serotonin-2C receptor G-protein-coupled, HTR2C, in porcine brain tissues. *Biochimie* 121 189–196. 10.1016/j.biochi.2015.12.011 26707647

[B42] Li Q. H. Nakadate K. Tanaka-Nakadate S. Nakatsuka D. Cui Y. Watanabe Y. (2004). Unique expression patterns of 5-HT2A and 5-HT2C receptors in the rat brain during postnatal development: western blot and immunohistochemical analyses. *J. Comp. Neurol.* 469 128–140. 10.1002/cne.11004 14689478

[B43] Lovinger D. M. (1997). Serotonin’s role in alcohol’s effects on the brain. *Alcohol Health Res. World* 21 114–120. 15704346 PMC6826824

[B44] Maas S. Kawahara Y. Tamburro K. M. Nishikura K. (2006). A-to-I RNA editing and human disease. *RNA Biol.* 3 1–9. 10.4161/rna.3.1.2495 17114938 PMC2947206

[B45] Marcucci R. Brindle J. Paro S. Casadio A. Hempel S. Morrice N. (2011). Pin1 and WWP2 regulate GluR2 Q/R site RNA editing by ADAR2 with opposing effects. *EMBO J.* 30 4211–4222. 10.1038/emboj.2011.303 21847096 PMC3199391

[B46] Martin C. B. Ramond F. Farrington D. T. Aguiar A. S. Jr. Chevarin C. Berthiau A. S. (2013). RNA splicing and editing modulation of 5-HT(2C) receptor function: relevance to anxiety and aggression in VGV mice. *Mol. Psychiatry* 18 656–665. 10.1038/mp.2012.171 23247076

[B47] Milatovich A. Hsieh C. L. Bonaminio G. Tecott L. Julius D. Francke U. (1992). Serotonin receptor 1c gene assigned to X chromosome in human (band q24) and mouse (bands D-F4). *Hum. Mol. Genet.* 1 681–684. 10.1093/hmg/1.9.681 1302605

[B48] Mohammad-Zadeh L. F. Moses L. Gwaltney-Brant S. M. (2008). Serotonin: a review. *J. Vet. Pharmacol. Ther.* 31 187–199. 10.1111/j.1365-2885.2008.00944.x 18471139

[B49] Mombereau C. Kawahara Y. Gundersen B. B. Nishikura K. Blendy J. A. (2010). Functional relevance of serotonin 2C receptor mRNA editing in antidepressant- and anxiety-like behaviors. *Neuropharmacology* 59 468–473. 10.1016/j.neuropharm.2010.06.009 20624407 PMC2946438

[B50] Morabito M. V. Abbas A. I. Hood J. L. Kesterson R. A. Jacobs M. M. Kump D. S. (2010). Mice with altered serotonin 2C receptor RNA editing display characteristics of Prader-Willi syndrome. *Neurobiol. Dis.* 39 169–180. 10.1016/j.nbd.2010.04.004 20394819 PMC2906772

[B51] Nishikura K. (2010). Functions and regulation of RNA editing by ADAR deaminases. *Annu. Rev. Biochem.* 79 321–349. 10.1146/annurev-biochem-060208-105251 20192758 PMC2953425

[B52] Niswender C. M. Copeland S. C. Herrick-Davis K. Emeson R. B. Sanders-Bush E. (1999). RNA editing of the human serotonin 5-hydroxytryptamine 2C receptor silences constitutive activity. *J. Biol. Chem.* 274 9472–9478. 10.1074/jbc.274.14.9472 10092629

[B53] Niswender C. M. Herrick-Davis K. Dilley G. E. Meltzer H. Y. Overholser J. C. Stockmeier C. A. (2001). RNA editing of the human serotonin 5-HT2C receptor. Alterations in suicide and implications for serotonergic pharmacotherapy. *Neuropsychopharmacology* 24 478–491. 10.1016/s0893-133x(00)00223-2 11282248

[B54] Nocjar C. Alex K. D. Sonneborn A. Abbas A. I. Roth B. L. Pehek E. A. (2015). Serotonin-2C and -2a receptor co-expression on cells in the rat medial prefrontal cortex. *Neuroscience* 297 22–37. 10.1016/j.neuroscience.2015.03.050 25818050 PMC4595040

[B55] Palacios J. M. Pazos A. Hoyer D. (2017). A short history of the 5-HT2C receptor: from the choroid plexus to depression, obesity and addiction treatment. *Psychopharmacology* 234 1395–1418. 10.1007/s00213-017-4545-5 28265714

[B56] Pandey S. C. Davis J. M. Pandey G. N. (1995). Phosphoinositide system-linked serotonin receptor subtypes and their pharmacological properties and clinical correlates. *J. Psychiatry Neurosci.* 20 215–225. 7786883 PMC1188687

[B57] Park E. Williams B. Wold B. J. Mortazavi A. (2012). RNA editing in the human ENCODE RNA-seq data. *Genome Res.* 22 1626–1633. 10.1101/gr.134957.111 22955975 PMC3431480

[B58] Pasqualetti M. Ori M. Castagna M. Marazziti D. Cassano G. B. Nardi I. (1999). Distribution and cellular localization of the serotonin type 2C receptor messenger RNA in human brain. *Neuroscience* 92 601–611. 10.1016/s0306-4522(99)00011-1 10408609

[B59] Sanders-Bush E. Breeding M. (1988). Putative selective 5-HT-2 antagonists block serotonin 5-HT-1c receptors in the choroid plexus. *J. Pharmacol. Exp. Ther.* 247 169–173. 3139864

[B60] Schellekens H. Clarke G. Jeffery I. B. Dinan T. G. Cryan J. F. (2012). Dynamic 5-HT2C receptor editing in a mouse model of obesity. *PLoS One* 7:e32266. 10.1371/journal.pone.0032266 22448217 PMC3308946

[B61] Schellekens H. De Francesco P. N. Kandil D. Theeuwes W. F. McCarthy T. van Oeffelen W. E. (2015). Ghrelin’s orexigenic effect is modulated via a serotonin 2C receptor interaction. *ACS Chem. Neurosci.* 6 1186–1197. 10.1021/cn500318q 25727097

[B62] Schellekens H. van Oeffelen W. E. Dinan T. G. Cryan J. F. (2013). Promiscuous dimerization of the growth hormone secretagogue receptor (GHS-R1a) attenuates ghrelin-mediated signaling. *J. Biol. Chem.* 288 181–191. 10.1074/jbc.M112.382473 23161547 PMC3537012

[B63] Schmidt H. D. McFarland K. N. Darnell S. B. Huizenga M. N. Sangrey G. R. Cha J. H. (2015). ADAR2-dependent GluA2 editing regulates cocaine seeking. *Mol. Psychiatry* 20 1460–1466. 10.1038/mp.2014.134 25349168 PMC4412769

[B64] Shirahase T. Watanabe Y. Tsujimura A. Kwak S. Yamamoto T. Kanamura N. (2018). Ethanol preference and drinking behavior are controlled by RNA editing in the nucleus accumbens. *Front. Behav. Neurosci.* 12:331. 10.3389/fnbeh.2018.00331 30697154 PMC6340988

[B65] Simmons M. Meador-Woodruff J. H. Sodhi M. S. (2010). Increased cortical expression of an RNA editing enzyme occurs in major depressive suicide victims. *Neuroreport* 21 993–997. 10.1097/WNR.0b013e32833f11c3 20802353

[B66] Sodhi M. S. Burnet P. W. Makoff A. J. Kerwin R. W. Harrison P. J. (2001). RNA editing of the 5-HT(2C) receptor is reduced in schizophrenia. *Mol. Psychiatry* 6 373–379. 10.1038/sj.mp.4000920 11443520

[B67] Spoida K. Masseck O. A. Deneris E. S. Herlitze S. (2014). Gq/5-HT2c receptor signals activate a local GABAergic inhibitory feedback circuit to modulate serotonergic firing and anxiety in mice. *Proc. Natl. Acad. Sci. U.S.A.* 111 6479–6484. 10.1073/pnas.1321576111 24733892 PMC4035925

[B68] Stamm S. Gruber S. B. Rabchevsky A. G. Emeson R. B. (2017). The activity of the serotonin receptor 2C is regulated by alternative splicing. *Hum. Genet.* 136 1079–1091. 10.1007/s00439-017-1826-3 28664341 PMC5873585

[B69] Tecott L. H. Sun L. M. Akana S. F. Strack A. M. Lowenstein D. H. Dallman M. F. (1995). Eating disorder and epilepsy in mice lacking 5-HT2c serotonin receptors. *Nature* 374 542–546. 10.1038/374542a0 7700379

[B70] Tohda M. (2014). Serotonin 2C receptor as a superhero: diversities and talents in the RNA universe for editing, variant, small RNA and other expected functional RNAs. *J. Pharmacol. Sci.* 126 321–328. 10.1254/jphs.14R06CR 25427431

[B71] Uchida H. Matsumura S. Okada S. Suzuki T. Minami T. Ito S. (2017). RNA editing enzyme ADAR2 is a mediator of neuropathic pain after peripheral nerve injury. *FASEB J.* 31 1847–1855. 10.1096/fj.201600950R 28126736

[B72] van de Wouw M. Stilling R. M. Peterson V. L. Ryan F. J. Hoban A. E. Shanahan F. (2019). Host microbiota regulates central nervous system serotonin receptor 2C editing in rodents. *ACS Chem. Neurosci.* 10 3953–3960. 10.1021/acschemneuro.9b00414 31415146

[B73] Wang Q. O’Brien P. J. Chen C. X. Cho D. S. Murray J. M. Nishikura K. (2000). Altered G protein-coupling functions of RNA editing isoform and splicing variant serotonin2C receptors. *J. Neurochem.* 74 1290–1300. 10.1046/j.1471-4159.2000.741290.x 10693963

[B74] Watanabe Y. Yoshimoto K. Tatebe H. Kita M. Nishikura K. Kimura M. (2014). Enhancement of alcohol drinking in mice depends on alterations in RNA editing of serotonin 2C receptors. *Int. J. Neuropsychopharmacol.* 17 739–751. 10.1017/S1461145713001545 24345557 PMC4220740

[B75] Weissmann D. van der Laan S. Underwood M. D. Salvetat N. Cavarec L. Vincent L. (2016). Region-specific alterations of A-to-I RNA editing of serotonin 2c receptor in the cortex of suicides with major depression. *Transl. Psychiatry* 6:e878. 10.1038/tp.2016.121 27576167 PMC5022077

[B76] Werry T. D. Loiacono R. Sexton P. M. Christopoulos A. (2008). RNA editing of the serotonin 5HT2C receptor and its effects on cell signalling, pharmacology and brain function. *Pharmacol. Ther.* 119 7–23. 10.1016/j.pharmthera.2008.03.012 18554725

[B77] Yoshimoto K. Komura S. (1989). Genetic differences in the effects of voluntary ethanol consumption on brain monoamine levels in inbred strains of mice, C57BL/6J, C3H/He and DBA/2Cr. *Alcohol Alcohol.* 24 225–229. 2569311

[B78] Yoshimoto K. McBride W. J. (1992). Regulation of nucleus accumbens dopamine release by the dorsal raphe nucleus in the rat. *Neurochem. Res.* 17 401–407. 10.1007/bf00969884 1356241

[B79] Yoshimoto K. Watanabe Y. Tanaka M. Kimura M. (2012). Serotonin2C receptors in the nucleus accumbens are involved in enhanced alcohol-drinking behavior. *Eur. J. Neurosci.* 35 1368–1380. 10.1111/j.1460-9568.2012.08037.x 22512261 PMC3490368

[B80] Zaidan H. Gaisler-Salomon I. (2015). Prereproductive stress in adolescent female rats affects behavior and corticosterone levels in second-generation offspring. *Psychoneuroendocrinology* 58 120–129. 10.1016/j.psyneuen.2015.04.013 25973567

[B81] Zaidan H. Ramaswami G. Golumbic Y. N. Sher N. Malik A. Barak M. (2018). A-to-I RNA editing in the rat brain is age-dependent, region-specific and sensitive to environmental stress across generations. *BMC Genomics* 19:28. 10.1186/s12864-017-4409-8 29310578 PMC5759210

[B82] Zaniewska M. Alenina N. Wydra K. Frohler S. Kusmider M. McCreary A. C. (2015). Discovering the mechanisms underlying serotonin (5-HT)2A and 5-HT2C receptor regulation following nicotine withdrawal in rats. *J. Neurochem.* 134 704–716. 10.1111/jnc.13192 26031442

[B83] Zhang Z. Shen M. Gresch P. J. Ghamari-Langroudi M. Rabchevsky A. G. Emeson R. B. (2016). Oligonucleotide-induced alternative splicing of serotonin 2C receptor reduces food intake. *EMBO Mol. Med.* 8 878–894. 10.15252/emmm.201506030 27406820 PMC4967942

